# Protective effects of hydrogen-rich saline in uncontrolled hemorrhagic shock

**DOI:** 10.3892/etm.2014.1572

**Published:** 2014-02-21

**Authors:** ZUNMIN DU, HAIPENG JIA, JING LIU, XIAOMIN ZHAO, YUN WANG, XUEJUN SUN

**Affiliations:** 1 Department of Hematology, The 88th Hospital of Chinese People’s Liberation Army, Tai’an, Shandong 271000, P.R. China; 2 Atherosclerosis Research Institute of Taishan Medical University, Tai’an, Shandong 271000, P.R. China; 3 Department of Diving Medicine, Faculty of Naval Medicine, The Second Military Medical University, Shanghai 200433, P.R. China

**Keywords:** uncontrolled hemorrhagic shock, inflammation factor, superoxide dismutase, malondialdehyde, antioxidant, oxidative damage, hydrogen-rich saline

## Abstract

Hydrogen is considered to be a novel antioxidant as it inhibits inflammation, removes oxygen-derived free radicals and reduces oxidative damage. This study investigated the effects of hydrogen-rich saline on plasma interleukin-6 (IL-6), tumor necrosis factor-α (TNF-α), superoxide dismutase (SOD) and malondialdehyde (MDA) in rats with uncontrolled hemorrhagic shock (UHS). The UHS model was induced by arterial bleeding and tail amputation. The rats were randomly divided into: Group A (sham-operated group), Group B [shock + intravenously (IV) injected saline], Group C (shock + IV-injected hydrogen-rich saline), Group D [shock + intraperitoneally (IP) injected saline] and Group E (shock + IP-injected hydrogen-rich saline). The survival rate 24 h after successful resuscitation was calculated. The mean arterial pressure and heart rate were recorded at 0, 30, 90 and 210 min. The plasma levels of IL-6, TNF-α, SOD and MDA were measured at 0, 90 and 210 min. The survival rate of each group was 100% and the hemodynamics among the experimental groups were not significantly different. At 90 and 210 min, the levels of IL-6, TNF-α and MDA in Groups C and E were lower than those of Groups B and D, while the SOD levels were higher than those of Groups B and D (P<0.01). At 90 min, the levels of IL-6, TNF-α and MDA in Groups B and C were lower than those of Groups D and E, respectively (P<0.01). Hydrogen-rich saline has anti-inflammatory and anti-oxidative effects in UHS. In conclusion, the results showed that itravenous injection of hydrogen-rich saline is more effective than intraperitonal injection.

## Introduction

Uncontrolled hemorrhagic shock (UHS) causes low perfusion of visceral organs, ischemia and hypoxemia of tissue, release of inflammatory factors, generation of oxygen-derived free radicals and oxidative damage of tissue, multiple organ dysfunction syndrome (MODS) and a low rate of treatment success. Previous studies have shown that the inhalation of 2% hydrogen or injection of hydrogen-saturated saline inhibits inflammation, selectively removes oxygen-derived free radicals and reduces oxidative damage (1–6), suggesting hydrogen-rich saline may play an important role in UHS. Therefore, we hypothesized that hydrogen-rich saline may have anti-inflammatory and anti-oxidative roles in the treatment of hemorrhagic shock. However, there are no studies concerning the effects of hydrogen-rich saline in UHS. The present study was designed to investigate and compare the effects of hydrogen-rich saline injected intravenously (IV) or intraperitoneally (IP) on the plasma levels of interleukin-6 (IL-6), tumor necrosis factor-α (TNF-α), superoxide dismutase (SOD) and malondialdehyde (MDA) in rats with UHS. The aim was to demonstrate the anti-inflammatory and anti-oxidative functions of hydrogen-rich saline on UHS and to discuss the mechanism of action of hydrogen-rich saline.

## Materials and methods

### Animals

Healthy male Wistar rats (n=50; 290–320 g) were provided by the Experimental Animal Center of Shandong University (Jinan, China). The study was approved by the Animal Ethics Committee of The 88th Hospital of Chinese People’s Liberation Army (Tai’an, China). All efforts were made to reduce the number of experimental animals used, to provide maximal relief of pain and to prohibit maltreatment of the animals.

### Preparation and preservation of hydrogen-rich saline

The hydrogen-rich saline was prepared according to the methods of a previous study (7). Briefly, hydrogen was dissolved in normal saline for 2 h under high pressure (0.4 MPa) to achieve the supersaturated level using a self-designed, hydrogen-rich water-producing apparatus. The hydrogen-rich saline (250 ml) was stored under atmospheric pressure at 4°C in an aluminum bag without dead volume. Hydrogen-rich saline was freshly prepared on a weekly basis to ensure a constant concentration of >0.6 mmol/l.

### Preparation of UHS model

Prior to the experiment, the rats were fed adaptively for one week at the Experimental Animal Center, Atherosclerosis Research Institute, Taishan Medical University (Tai’an, China). The feeding environment was maintained at a temperature of 25±2°C, humidity of 55% and with light-dark alternation at 12-h intervals. The rats were provided food and water *ad libitum*. Immediately prior to the experiment, the rats were deprived of food for 12 h and of water for 8 h. Sodium pentobarbital (1%; 4 mg/100 g body weight) was injected into the thigh muscle of the posterior limb for anesthesia at a fixed rate. Two tubes were inserted into the two femoral arteries. One tube was connected to a survival rate physiological signal recorder to monitor mean arterial pressure (MAP) and heart rate (HR), and the other tube was used to allow bleeding and to obtain blood samples. A third tube was inserted into the left femoral vein for injection of heparinized saline (50 U/100 g body weight) and intravenous transfusion. The UHS rat model was induced through an artery and tail amputation, according to the methods of a previous study (8). Briefly, the animals were stabilized for 10 min after anesthesia and intubation, following which the experimental timing commenced. Within 15 min, intermittent bleeding was conducted through the tube inserted into the femoral artery to maintain the MAP at 45 mmHg and the MAP was maintained at this level through adjustment of the blood loss quantity for 30 min. The blood obtained from the femoral artery was stored in a heparinized 10-ml sterile injector for blood retransfusion. At 45 min, 75% of the tail was amputated to cause active bleeding and the blood was collected in a heparinized sterile tube.

### Experimental stages and groups

The 50 rats were divided into five groups of 10 rats each. For Group A (sham-operated group), only anesthesia and intubation were implemented, the MAP and HR were monitored and blood samples were obtained. For Groups B and C, plain or hydrogen-rich saline was injected IV, respectively, at 30 min through the tube inserted into the femoral vein at a rate of 3–4 ml/h to maintain the MAP at 60±5 mmHg. For Groups D and E, 10 ml/100 g body weight of plain or hydrogen-rich saline was injected IP, respectively, at 30 min to maintain the MAP at 60±5 mmHg. At 90 min, Yunnan white drug-powder was applied externally to the end of the tails of the rats and the tail end was bound up with sterile gauze for hemostasis and blood transfusion. The MAP was raised to ≥90 mmHg by adjusting the transfusion rate. In the observation period, the rats were carried back to cages for individual feeding, with free access to water and food.

### Survival rate

The survival rate of the experimental rats was calculated as: Number of rats surviving at 24 h after successful resuscitation/number of rats in the same group ×100%.

### Hemodynamic monitoring

The multi-lead physiological signal recorder (type MP150; Biopac Systems, Inc., Goleta, CA, USA) was used for real-time monitoring of the MAP and HR. The MAP and HR of the rats in each group at 0, 30, 90 and 210 min were analyzed. MAP and HR were recorded during the observation period in freely moving animals.

### Determination of plasma IL-6, TNF-α, SOD and MDA levels

The arterial blood (0.5 ml) was obtained from the tube inserted in the femoral artery of rats at 0, 90 and 210 min and maintained at 4°C for 2 h. Subsequently, the blood was centrifuged for 10 min at 650 × g, and the serum was refrigerated at −80°C for subsequent experiments. Enzyme-linked immunosorbent assay kits (Shanghai Lengton Bioscience Co., Ltd., Shanghai, China) were used to determine the plasma levels of IL-6, TNF-α, SOD and MDA according to the manufacturer’s instructions. Following blood sampling, a corresponding amount of saline or hydrogen-rich saline was immediately supplied through the femoral vein.

### Statistical analysis

The software SPSS, version 13.0 for windows (SPSS, Inc., Chicago, IL, USA) was used for data analysis. Data are expressed as the mean ± standard deviation. For the overall comparison of the differences at different time points and in different groups, an analysis variance designed for repeated measures was used. For within-group comparisons at the same time point, one-way analysis of variance was used, and the Student-Newman-Keuls post-hoc test was used for pairwise comparisons. P<0.05 was considered to indicate a statistically significant difference.

## Results

### General

The survival rate of the rats in each group was 100%. The weight of the rats in each group was comparable, and no significant differences in the blood loss, blood transfusions or infusion volume were observed (Table I). All these factors were controlled, thus no differences were observed.

### Hemodynamics

As shown in Tables II and III, at 0 min, the MAP was comparable among the five groups. At 30 min, the MAP in the four experimental groups (Groups B-E) was significantly lower than that of the sham-operated group (P<0.01), but was not significantly different among the four experimental groups, showing that the UHS model was established successfully. A significantly lower MAP compared with that of the normal did not indicate UHS. At 30 and 90 min, no significant difference among the experimental groups in the MAP or HR was observed, suggesting that the hydrogen-rich saline had no marked influence on the resuscitation of the blood pressure and HR, regardless of whether it was injected IV or IP.

### Hydrogen-rich saline inhibits the generation and release of IL-6 and TNF-α in UHS rats

As shown in Table IV, the IL-6 levels in each experimental group gradually increased as the recovery increased. At 90 and 210 min, the IL-6 levels in Group D were significantly higher when compared with those of Groups B and E, and the IL-6 levels in Groups B and E were higher when compared with those of Group C (P<0.01).

As shown in Table V, the TNF-α levels in the four experimental groups (Groups B-E) increased initially and then decreased slightly. In these four groups, the TNF-α levels at 90 min were significantly higher than those at 210 min and were significantly greater than those in the sham-operated group at the two time-points (P<0.01 for all comparisons). At 90 min, the TNF-α levels in Groups B and E were significantly higher than those in Group C, and the TNF-α levels in Group D were significantly higher than those in Groups B and E (P<0.01). At 210 min, the TNF-α levels in Group B were higher than those in Group C, and the TNF-α levels in Group D were higher than those in Groups B and E (P<0.01 for all comparisons). However, no significant difference was observed between the levels in Groups C and E.

### Hydrogen-rich saline reduces the consumption of SOD and production of MDA in UHS rats

The SOD levels gradually decreased as the recovery time increased (Table VI). At 90 and 210 min, the SOD levels in Groups C and E were higher than those in Groups B and D, respectively (P<0.01 for each comparison). However, no significant differences were observed between Groups C and E or Groups B and D.

The MDA levels in each experimental group gradually increased as the recovery time increased (Table VII). At 90 and 210 min, the MDA levels in each experimental group were increased significantly from the baseline (0 min; P<0.01). At the two time points, the MDA levels in Groups C and E were lower than those in Groups B and D, respectively (P<0.01). Additionally, at 90 and 210 min, the MDA levels in Groups B and C were significantly lower than those in Groups D and E, respectively (P<0.01).

## Discussion

Hemorrhagic shock may cause a series of responses, including the release of inflammatory factors such as TNF and IL-6 and oxidative damage, while inflammatory factors and oxygen-derived free radicals play an important role in the development of shock (9,10). In 1986, Tracey *et al* mimicked the pathological changes in hemodynamics and the injuries of cells and tissues in infectious shock using rats injected with recombinant human TNF-α (11). Additionally, Qiu *et al* identified the combination of TNF-α with mTNF-1R in the inflammatory reaction (12). IL-6 is generated by monocyte-macrophage cells, T-helper type 2 cells, endothelial cells and numerous other types of cell, and has a close association with the severity and lethality of systemic inflammatory response syndrome and MODS. IL-6 not only activates and poisons vascular endothelial and inflammatory cells, but also induces the synthesis of acute-phase proteins, catalyzes and amplifies the inflammatory reaction and toxicity, and causes damage to histiocytes and MODS (13). Active oxygen-free radicals attack the biological membrane of unsaturated fatty acids, causing lipid peroxidation into MDA. Thus, the content of MDA is a measure of the extent of lipid peroxidation damage (14). SODs are a group of heavy metal enzymes that reduce the levels of oxygen-derived free radicals and moderate the inflammatory reaction resulting from shock and ischemia-reperfusion injury. Tan *et al* (15) and Rhee *et al* (16) reported that, in a hemorrhagic shock animal model, the oxygen-derived free radical scavenger SOD reduces fatalities. In the present study, it was found that the levels of IL-6, TNF-α and MDA were increased while the levels of SOD were reduced in UHS rats compared with those of sham-operated rats, suggesting the existence of the inflammatory response in UHS and oxidative damage, and this result is consistent with that of previous studies (17).

In 2007, Ohsawa *et al* demonstrated that hydrogen significantly improves brain ischemia-reperfusion injury and increases the SOD levels in animals by selectively neutralizing hydroxyl radicals and peroxynitrite anions, which are the most important causes of oxidative damage (6), suggesting that hydrogen may improve the levels of SOD activity in the endogenous antioxidant system and reduce the levels of activated oxygen-derived free radicals. In a study of the protective action of hydrogen against radiation damage in mice, Qian *et al* showed that IP injection of hydrogen-saturated saline prior to the delivery of radiation improves the SOD levels in the plasma (18). Findings of those studies are consistent with the results of the present study that hydrogen increases the levels of MDA but decreases the levels of SOD, suggesting the protective effect of hydrogen in UHS is through an anti-oxidative effect. However, in the present study, a difference in the survival rate among the groups of rats was not observed. Xie *et al* reported that 2% hydrogen inhalation at 1 and 6 h after establishment of a sepsis model increased the survival rate of mice with moderate and severe blood poisoning and attenuated organ damage (19). The discrepancy may be partially due to the mild hemorrhagic shock in the present study, which is supported by the result that no differences in the MAP and HR were found among the different groups. In addition, the present study indicated that IV-injected hydrogen has an improved effect compared with that of IP-injected hydrogen, which is consistent with the results of a previous study (20), indicating that the effect of hydrogen may be associated with its pharmacokinetics.

The mechanism of the anti-oxidative effect of hydrogen in hemorrhagic shock may be associated with its anti-inflammatory effect. This hypothesis is supported by previous studies (6,18) and the result of the present study that hydrogen decreases the plasma levels of IL-6 and TNF-α. In addition, Xu *et al* demonstrated that hydrogen inhibits the infiltration of partial neutrophilic granulocytes, reduces the mRNA levels of TNF-α in activated macrophages and inhibits TNF-α secretion by macrophages (21). In models for intestinal transplantation (22) and intestinal ischemia/reperfusion (23), hydrogen relieves the damage of the intestinal implant, reduces the levels of IL-1β and IL-6 in the muscular layer of the implant and lung tissue, and reduces the mRNA levels of chemotactic factors and the infiltration of neutrophilic granulocytes in the lung and intestinal tissues. These results suggested an anti-inflammatory effect of hydrogen.

In summary, the present study has shown that hydrogen has a protective effect in UHS which is associated with its anti-inflammatory and anti-oxidative actions. In addition, IV-administered hydrogen has an improved effect compared with that of IP-administered hydrogen.

## Figures and Tables

**Table I:** Characteristics of the rats in the different groups (n=10/group; mean ± SD).

Group	Weight (g)	Blood loss (ml)	Blood transfusions (ml)	Infusion volume (ml)
A	302.60±8.02	0.5	0	0
B	304.10±9.45	6.85±0.27	6.24±0.40	13.91±1.64
C	308.90±4.48	6.87±0.29	6.34±0.34	14.55±1.42
D	302.60±8.02	7.07±0.19	6.55±0.16	14.30±1.49
E	303.10±9.75	6.88±0.36	6.48±0.33	13.05±1.17
F value	1.03	1.31	2.04	2.09
Pr>F	>0.05	>0.05	>0.05	>0.05

**Table II:** Changes in the MAP of the rats in the different groups at four time points (n=10/group; mean ± SD, mmHg).

	Time after injury (min)			
	
Group	0	30	90	210
A	128.00±1.79	129.50±3.61	128.16±3.82	124.00±3.10
B	128.67±5.20	39.83±1.834^a^	62.00±3.58^a^	99.67±4.08^a^
C	126.83±11.41	40.83±1.94^a^	59.50±2.59^a^	108.50±9.50^a^
D	123.17±6.11	39.50±1.87^a^	61.17±2.99^a^	112.17±9.80^a^
E	131.00±9.30	41.67±2.65^a^	60.33±2.16^a^	107.83±11.36^a^
F value	0.87	1545.21	572.24	6.88
Pr>F	>0.05	<0.01	<0.01	<0.01

a P<0.01 vs. Group A.

MAP, mean arterial pressure.

**Table III:** Changes in the HR of the rats in the different group at four time points (n=10/group; mean ± SD, beats/min).

	Time after injury (min)			
	
Group	0	30	90	210
A	485.67±27.37	467.50±8.76	461.33±16.13	492.50±41.29
B	469.33±13.31	489.67±13.65	476.00±4.90	451.67±36.15
C	475.17±18.76	492.17±16.52	472.67±19.19	456.67±41.00
D	479.17±44.29	497.00±37.94	463.17±11.92	478.83±22.61
E	476.00±26.86	496.83±26.78	455.50±32.65	477.50±30.57
F value	0.27	1.66	1.15	1.40
Pr>F	>0.05	>0.05	>0.05	>0.05

HR, heart rate.

**Table IV:** Changes in the IL-6 levels of the rats in the in different groups at three time points (n=10/group; mean ± SD, ng/l).

	Time after injury (min)		
	
Group	0	90	210
A	72.24±15.54	74.58±13.79	75.21±14.17
B	71.91±14.95	179.13±12.86^a^,^c^	268.93±8.55^a^,^c^
C	74.41±12.72	140.78±7.61^a^,^b^,^d^	233.07±6.28^a^,^b^,^d^
D	71.24±15.36	209.86±10.08^a^,^b^,^d^	308.07±14.57^a^,^b^,^d^
E	71.74±12.81	170.95±9.85^a^,^c^	270.11±10.30^a^,^c^
F value	0.04	129.37	393.85
Pr>F	>0.05	<0.01	<0.01

a P<0.01 vs. sham-operated group;

b P<0.01 vs. Group B;

c P<0.01 vs. Group D;

d P<0.01 vs. Group E.

IL-6, interleukin-6.

**Table V:** Changes in the TNF-α levels of the rats in the different groups at three time points (n=10/group; mean ± SD, ng/l)

	Time after injury (min)		
	
Group	0	90	210
A	25.07±3.54	28.07±2.43	26.40±3.87
B	27.40±4.18	157.27±6.39^a^,^c^,^e^	136.22±7.17^a^,^c^
C	26.40±3.14	134.61±4.68^a^,^b^,^d^,^e^	108.38±5.60^a^,^b^
D	28.57±1.55	165.29±4.00^a^,^b^,^e^	146.01±4.67^a^,^b^
E	25.40±4.06	145.56±3.77^a^,^c^,^e^	110.99±9.23^a^,^c^
F value	1.06	953.89	325.60
Pr>F	>0.05	<0.01	<0.01

a P<0.01 vs. sham-operated group;

b P<0.01 vs. Group B;

c P<0.01 vs. Group D;

d P<0.01 vs. Group E;

e P<0.01 vs. 210 min.

TNF-α, tumor necrosis factor-α

**Table VI:** Changes in the SOD levels of the rats in the different groups at three time points (n=10/group; mean ± SD, ng/l).

	Time after injury (min)		
	
Group	0	90	210
A	3.71±0.21	3.65±0.20	3.71±0.21
B	3.64±0.18	1.89±0.05^a^	1.17±0.19^a^
C	3.80±0.16	2.37±0.07^a^,^b^	1.78±0.09^a^,^b^
D	3.78±0.12	1.81±0.09^a^	1.19±0.12^a^
E	3.76±0.17	2.22±0.47^a^,^c^	1.63±0.08^a^,^c^
F value	0.82	61.28	309.17
Pr>F	>0.05	<0.01	<0.01

a P<0.01 vs. Group A;

b P<0.01 vs. Group B;

c P<0.01 vs. Group D.

SOD, superoxide dismutase.

**Table VII:** Changes in the MDA levels of the rats in the different groups at three time points (n=10/group; mean ± SD, nmol/ml).

	Time after injury (min)		
	
Group	0	90	210
A	3.31±0.15	3.32±0.14	3.41±0.18
B	3.27±0.07	5.33±0.10^a^,^c^	7.30±0.07^a^,^c^
C	3.34±0.15	4.55±0.23^a^,^b^	6.27±0.07^a^,^b^
D	3.34±0.15	5.88±0.05^a^,^b^	7.53±0.08^a^,^b^
E	3.30±0.10	5.13±0.07^a^,^c^	7.12±0.05^a^,^c^
F value	0.35	312.23	1663.08
Pr>F	>0.05	<0.01	<0.01

a P<0.01 vs. Group A;

b P<0.01 vs. Group B;

c P<0.01 vs. Group D.

MDA, malondialdehyde.
